# The Guanine-Based Purinergic System: The Tale of An Orphan Neuromodulation

**DOI:** 10.3389/fphar.2016.00158

**Published:** 2016-06-17

**Authors:** Valentina Di Liberto, Giuseppa Mudò, Roberta Garozzo, Monica Frinchi, Víctor Fernandez-Dueñas, Patrizia Di Iorio, Renata Ciccarelli, Francesco Caciagli, Daniele F. Condorelli, Francisco Ciruela, Natale Belluardo

**Affiliations:** ^1^Department of Experimental Biomedicine and Clinical Neurosciences, University of PalermoPalermo, Italy; ^2^Department of Biomedical and Biotechnological Sciences, Unit of Medical Biochemistry, University of CataniaCatania, Italy; ^3^Department of Pathology and Experimental Therapeutics, Faculty of Medicine, Bellvitge Biomedical Research Institute, Institute of Neurosciences, University of BarcelonaBarcelona, Spain; ^4^Department of Medical, Oral and Biotecnological Sciences, University of Chieti-PescaraChieti, Italy

**Keywords:** guanine-based purines, guanosine, neuroprotection, synaptic plasticity, purinergic receptors, adenosine

## Abstract

Guanine-based purines (GBPs) have been recently proposed to be not only metabolic agents but also extracellular signaling molecules that regulate important functions in the central nervous system. In such way, GBPs-mediated neuroprotection, behavioral responses and neuronal plasticity have been broadly described in the literature. However, while a number of these functions (i.e., GBPs neurothophic effects) have been well-established, the molecular mechanisms behind these GBPs-dependent effects are still unknown. Furthermore, no plasma membrane receptors for GBPs have been described so far, thus GBPs are still considered orphan neuromodulators. Interestingly, an intricate and controversial functional interplay between GBPs effects and adenosine receptors activity has been recently described, thus triggering the hypothesis that GBPs mechanism of action might somehow involve adenosine receptors. Here, we review recent data describing the GBPs role in the brain. We focus on the involvement of GBPs regulating neuronal plasticity, and on the new hypothesis based on putative GBPs receptors. Overall, we expect to shed some light on the GBPs world since although these molecules might represent excellent candidates for certain neurological diseases management, the lack of putative GBPs receptors precludes any high throughput screening intent for the search of effective GBPs-based drugs.

## Introduction

Guanine-based purines (GBPs), including the nucleotides guanosine 5′-triphosphate (GTP), guanosine 5′-diphosphate (GDP) and guanosine 5′-monophosphate (GMP), the nucleoside guanosine (GUO) and the nucleobase guanine (GUA) have been traditionally characterized as modulators of intracellular processes, especially considering their role in G protein dependent signal transduction. In the last two decades, GBPs have been also shown to exert extracellular effects and to act as neuromodulator pleiotropic molecules affecting several cellular processes, including growth, differentiation and survival, in both the central and the peripheral nervous system (CNS and PNS, respectively; Schmidt et al., 2007; Rathbone et al., 2008). However, despite accumulated experimental evidences supporting their extracellular role, putative receptor sites have not been characterized, thus GBPs are still orphan neuromodulators. In this article, apart from reviewing recent data illustrating the involvement of GBPs in the nervous system, we especially discuss the new hypothesis concerning the existence of putative GBPs receptors.

## Purinergic System and “Purinome”

The term “purinergic system” usually refers to a complex system composed by purines bases, such as adenine (ADA) and GUA, their corresponding nucleotides adenosine 5′-triphosphate (ATP) and GTP, adenosine 5′-diphosphate (ADP) and GDP, adenosine 5′-monophosphate (AMP) and GMP, and nucleosides adenosine (ADO), inosine (INO) and GUO. In addition, the purinergic system includes the metabolites xanthine (XAN), hypoxanthine (HYPO) and uric acid (UA), together with the receptors, transporters and enzymes involved in purinergic transmission (Schmidt et al., 2007). Classically, purinergic signaling has been proposed to be regulated by the interaction of natural purines with a large number of specific own receptors, by a complex machinery assuring cell signaling transmission as well as by purine clarification from the extracellular medium by ecto-enzymes and/or bidirectional carriers (see below). Noteworthy, an emerging theory postulates that a broad-spectrum of direct/indirect interactions also exist within a cooperative synergic network, defined as “purinome” (Volonte and D’Ambrosi, 2009). The functions of the purinome involve the interaction and heteromerization between purinergic and other receptors. Interestingly, it is nowadays widely accepted that direct receptor-receptor interactions (i.e., oligomerization) permit to integrate information from different sources, thus providing a fine and complex control over various regulatory mechanisms (Ferre, 2007; Borroto-Escuela et al., 2012; Fuxe et al., 2012; Di Liberto et al., 2014). Regarding purinergic receptors, it has been shown, for instance, that adenosine 1 receptor (A_1_R) heterodimerizes with adenosine 2A receptor (A_2A_R), and also with dopamine D1, metabotropic glutamate mGlu1α, cannabinoid CB1 and adrenergic β1-and β2-adrenergic receptors, leading to diverse functional outcomes (Gines et al., 2000; Ciruela et al., 2001, 2006a,b; Chandrasekera et al., 2013). Recently, based on experimental evidence, Borroto-Escuela et al. (2014) proposed a network representation of G-protein coupled receptors (GPCR) heterodimers in the brain, in which A_1_R receptor emerges as a hub receptor forming 11 different heteroreceptor complexes.

## Extracellular Purines: Their Release and Re-Uptake.

The extracellular concentration of purines is dependent on the amount of released purines, on the efficiency of re-uptake mechanisms and on the activity of extracellular metabolizing enzymes. Purines can be released to the extracellular space by both neurons and glial cells, under physiological and, even more interestingly, in pathological conditions. Adenine-based purines (ABPs), for example ATP, can be released from neuronal presynaptic terminals and glial cells (Burnstock et al., 1972; Wagner et al., 1978; Burnstock, 2006), where they can be used as substrate for several ectonucleotidases (Zimmermann, 1996; Bonan, 2012; Zimmermann et al., 2012), which generate multiple breakdown products (ADP, AMP, and ADO) able to activate different receptors and to trigger several effects (Zimmermann, 2011).

GTP is co-stored together with ATP in neuronal synaptic vesicles from which it is released in association with ATP by exocytosis (Wagner et al., 1978; Santos et al., 2006). On the other hand, ATP is not involved in the outflow of GTP from glial cells. Nevertheless, GTP and its di- and mono-phosphate derivatives, together with the corresponding adenine-based counterparts, are substrates and thus competitors of the same ecto-nucleotidases while neurons seem to be able to release very small amounts of ADO *per se*, the presence of ADO outside glial cells is mainly due to the extracellular metabolism of released ATP. There are some peculiar differences concerning the other ADA- and GUA-based nucleosides, INO and GUO. Although these nucleosides derive from the hydrolysis of extracellular Inosine monophosphate (IMP)/GMP or from ADO deamination (for INO), they would also be constitutively released from neurons, astrocytes, C6 or U373 glioma cells, microglial cells or SH-SY5Y neuroblastoma cells (Ballerini et al., 2005, 2006a,b; Giuliani et al., 2012c). However, non-physiological and relatively extreme stimulations (e.g., seizure activity, hypoxia/hypoglycemia, and superfusion with high K^+^) seem to be required to elicit ADO eﬄux from glial cells; accordingly, in such conditions extracellular ADO would be that escaping from uptake and metabolism, thus representing a tissue overflow rather than a release *per se* (Di Iorio et al., 1998).

Finally, regarding purine metabolites such as HYPO, GUA, and XAN, which can be found in the medium of cultured glial cells and in the fluid from super-fused brain slices (Rathbone et al., 1999; Zamzow et al., 2008), it was thought during several years that they were uniquely transported outside the cells by specific transporters (NBTs), different from those for nucleosides (ENTs) (Sinclair et al., 2000; Dos Santos-Rodrigues et al., 2014). Thus, their presence outside of cells was considered, like for ADO, the result of cell overflow of substances to be eliminated (Parkinson et al., 2011). However, these purine metabolites would still be present in the extracellular milieu of cells treated with inhibitors of transporters, suggesting that they would also derive from the extracellular metabolism of nucleosides (Rathbone et al., 1999; Jiang et al., 2008b; Giuliani et al., 2012a; Caciagli et al., 2014).

Taken together, the previous findings indicate that the systems of ABPs and GBPs are physically and functionally present outside cells, where they operate simultaneously. In addition some peculiarities deserve to be emphasized. For instance: (i) the extracellular levels of GBPs are about 2-3-fold higher than those of their ADA-based counterparts (Ciccarelli et al., 1999); (ii) the metabolism of ADA and GUA nucleotides maintains constant the proportions of the components of the two systems; however, there are some differences in the respective nucleosides, such as the presence of only one GUA (GUO) instead of the two ADA nucleosides (ADO and INO), or the different affinities of these compounds for the trans-membrane transport systems, as well as for the enzymes deputed to their metabolism; and (iii) compared to ADO, the extracellular levels of GUO remain particularly elevated after an ischemic insult, a fact that has been shown both in cultured astrocytes (Ciccarelli et al., 1999) and in a model *in vivo* of focal cerebral ischemia (Uemura et al., 1991). To end with, it is important to underline that it is not an easy task to determine the exact concentrations of extracellular GBPs. Thus, the most common analytical assays, such as HPLC or capillary electrophoresis, do not permit to differentiate some peaks of ADA and GUA compounds, which are often overlapped (e.g., HYPO and GUA), thus their quantification cannot be carried out properly. Hence, to ameliorate the knowledge of GBPs system it is still necessary to improve the analytical methods, in order to separate these compounds from the ABPs counterparts (Ito et al., 2000; Stentoft et al., 2014).

## Release of Enzymes Metabolizing Purines

It is well known that purine metabolism is mainly oriented to preserve, by multiple pathways, the levels of triphosphate nucleotides. The enzyme system regulating the homeostasis of extracellular purines largely corresponds to that responsible for the metabolism and salvage of intracellular ABPs and GBPs. Indeed, it is well known that a broad spectrum of membrane-bound nucleotidases contribute to the breakdown of extracellular nucleotides (Zimmermann, 1996; Zimmermann et al., 2012). Moreover, it has also been recently reported that some soluble nucleotide kinases are released from cells and contribute to restore ATP levels when the extracellular amount of AMP becomes elevated (Yegutkin, 2014). In contrast, no ecto-enzymes seem to be involved in the catabolism of extracellular ADA- and GUA-based nucleosides, which would likely and only depend on some actively released soluble enzymes.

A few number of reports have indicated that some enzymes of the metabolism of purine nucleosides, such as ADO deaminase, purine nucleoside phosphorylase (PNP), Guanase and Xanthine Oxidase (XO), are detectable in plasma (Roberts and Newton, 2004; Roberts et al., 2004; Karabulut et al., 2005; Chittiprol et al., 2007; Lopez-Cruz et al., 2014; Giuliani et al., 2016). Furthermore, some of these enzymes have been proposed as potential diagnostic or prognostic markers for different pathological conditions. For instance, PNP plasma levels have been associated with the malignant degree and the diffusion capability of some tumors, such as the melanoma or pancreatic adenocarcinoma (Roberts et al., 2004; Vareed et al., 2011); whereas XO activity, which is physiologically low in plasma and increases after ischemic events, has been considered predictive for cardiovascular disease, independently of uric acid plasma levels (Gondouin et al., 2015; Lopez-Cruz et al., 2014). Nevertheless, it is still not sufficiently elucidated the origin of these plasmatic enzymes, the mechanisms by which they are released from cells and their activity in the metabolism of the extracellular purine nucleosides, importantly in the proximity of membrane purine receptors.

Recently, it has been observed, either in cell lines or in primary cultures of neural cells, that glial cells, especially microglia, but not neurons are able to constitutively release PNP in the culture medium. Extracellular K^+^ or exogenous ATP, but no other types of cell stimulation, such as LPS or interferon-γ, enhance, in a dose-dependent manner and via the lysosomal system, PNP release in the culture medium, where the enzyme is detectable both as soluble fraction and inside of membrane-derived vesicles (Polazzi et al., 2011). Interestingly, similar results have been shown investigating Guanase (Caciagli et al., 2014). These findings suggest that at least glial cells release the key enzymes of the metabolism of ABP and GBP nucleosides into the corresponding bases, which may act as potent cell damaging pro-inflammatory agents via reactive oxygen species (ROS) production. Therefore, the complex interplay between the activity of these enzymes and the transport systems of nucleosides across membranes may control the cell-specific homeostasis of extracellular purines as well as their signaling.

In physiological conditions, the activity of these kinds of enzymes controls the production of purine bases maintaining their levels in a range of “limited ability” to generate ROS. This delicate equilibrium seems to be lost during degenerative processes, which are often associated with chronic inflammatory, or in hypoxic, events. Hypoxia, for example, by inducing the over-expression/activity of 5′-nucleotidase and a concomitant blockade of the PNP activity, accelerates the hydrolysis of the nucleotides, thus causing a consequent accumulation of intra- and extra-cellular nucleosides, especially GUO (Ciccarelli et al., 1999). Similarly, during post-ischemic reperfusion, the functional block of the PNP seems to be not sufficient, resulting in the formation of large amounts of purine bases, whose catalytic oxidation, mediated by XO, produces ROS that through the activation of Nuclear factor kappa B (NF-kB) produce pro-inflammatory cytokines (Lorne et al., 2008).

Noteworthy, further emerging evidences support a relevant patho-physiological significance for these enzymes, which may have also some functions independent from their enzymatic activity. Among these enzymes, it is important to mention Nucleotidase II (Tozzi et al., 2013), which is currently being investigated (looking for its putative role outside cells) in our laboratory. This enzyme seems to interact with the ice protease-activating factor (IPAF) in order to regulate its folding and conformation, thus acting as a sensor of the global health state of the cell, and capable of regulating its death (Cividini et al., 2015). On its own, Guanase, named also cypin, plays a trophic role on the development of brain by regulating the dendritic arborization and neuronal morphology. In addition, Guanase seems to be involved in the mechanisms responsible of liver transplant rejection (Fernandez et al., 2010). Overall, the above-mentioned non-enzymatic activities of enzymes involved in purine nucleosides may be even more attractive as potential targets for developing new drugs.

## Purinergic Receptors

The present review is mainly focused on GBPs functions. However, we first provide a short overview of ABPs receptors, in order to contextualize the current knowledge of GBPs receptors into the purinergic field.

### ABPs Receptors: Long Story Made Short

ABPs play a pivotal role in the modulation of neurotransmission and neurotrophism acting on two different families of receptors: P1, activated by ADO, and P2, activated by ATP/ADP nucleotides. P1 receptor family includes four different subtypes (A_1_R, A_2A_R, A_2B_R, and A_3_R) of GPCRs. A_1_Rs and A_3_Rs are typically coupled to Gi proteins and thus inhibit adenylyl cyclases, whereas A_2A_Rs and A_2B_Rs are coupled to Gs proteins and increase the production of cyclic AMP (cAMP) (Zimmermann, 2011). P1 receptors are expressed in neurons, astrocytes, oligodendrocytes and microglia and their stimulation activates multiple functions, such as synaptic plasticity (Dare et al., 2007; Burnstock et al., 2011a,b; Zimmermann, 2011; Del Puerto et al., 2013). The P2 receptors are subdivided in two different subfamilies: ionotropic and metabotropic receptors (P2XRs and P2Yrs, respectively). The P2XRs subfamily includes seven different subtypes, which are mostly expressed by neurons and astrocytes, and in a lesser extent by oligodendrocytes, Schwann cells, and microglia. ATP mediated activation of P2XRs enhances rapid changes in membrane potential by increasing Na^+^, K^+^, and Ca^2+^permeability (North, 2002; Roberts et al., 2006; Del Puerto et al., 2013). Thus, they are involved in the regulation of fast synaptic transmission, synaptic plasticity, and fast neuron–glia signaling (Silinsky et al., 1992; Pankratov et al., 1998, 2002, 2009; Burnstock et al., 2011a; Lalo et al., 2011a,b). Conversely, the P2Y subfamily is composed by eight different P2YRs subtypes (Burnstock, 2007b). Interestingly, while P2Y_1_R, P2Y_2_R, P2Y_4_R, P2Y_6_R, and P2Y_11_R are coupled to Gq proteins and activate phospholipase C (PLC), the P2Y_12_R, P2Y_13_R, and P2Y_14_R are coupled to Gi proteins and inhibit adenylyl cyclases (Abbracchio et al., 2006). Overall, most of these receptors are expressed in neurons and glial cells, and are involved in bi-directional neuron–glia communication, thus exerting long-term effects on proliferation, neurogenesis, differentiation, migration and apoptosis (Burnstock, 2007a; Neary and Zimmermann, 2009; Verkhratsky et al., 2009; Del Puerto et al., 2013). For a more detailed introduction to ABPs receptors biology several well-documented reviews are available (Burnstock, 2007a,b; Burnstock et al., 2011a,b).

### GBPs Receptors: Short Story Made Long

At present, despite the plethora of experimental evidences pointing to the existence of putative GBPs receptors in the brain, a specific receptor has not been still identified; accordingly, GBPs are orphan neuromodulators. However, a number of cellular effects of GBPs might only be explained by the activation of different extracellular binding sites, since most of them still persist in the presence of inhibitors of GBPs transporters (Gysbers and Rathbone, 1992; Di Iorio et al., 2004). Actually, specific binding sites for GTP in PC12 cells (Gysbers et al., 2000) and the presence of a single high affinity binding site for [^3^H]-GUO in rat brain membranes (Traversa et al., 2002, 2003) have been described, supporting the possible existence of GBPs transmembrane receptors. GUO binding on rat membranes is saturable, reversible and is not displaced by other naturally occurring purines, such as ADO, HYPO, XAN, caffeine, theophylline, GDP, GMP, and ATP, thus suggesting that this binding site does not involve receptors for ABPs (Traversa et al., 2002). Indeed, GUO or GTP do not bind with high-affinity to ABPs receptors (Muller and Scior, 1993). The available data is in agreement with the fact that GBPs, in particular GUO, bind to metabotropic receptors and that many effects of GBPs are mediated through G protein-dependent signaling pathways; it has been shown, for instance, that the pertussis toxin-mediated inhibition of Gi/Go-protein reverses some of the effects of GUO on cell viability and glutamate uptake in hippocampal slices (Dal-Cim et al., 2013). Furthermore, by monitoring the binding of a non-hydrolysable-labeled GTP, it has been demonstrated that GUO is able to activate a putative not yet identified GPCR, different from the well-characterized ADO receptors, in rat brain membranes, (Volpini et al., 2011). Interestingly, we were able to obtain similar results, since we determined the activation of a putative unknown GPCR for GUO by means of binding experiments and *in situ* autoradiography of [^35^S]GTPγS, respectively, in membranes and slices from rat brain (Grillo et al., 2012).

**FIGURE 1:**
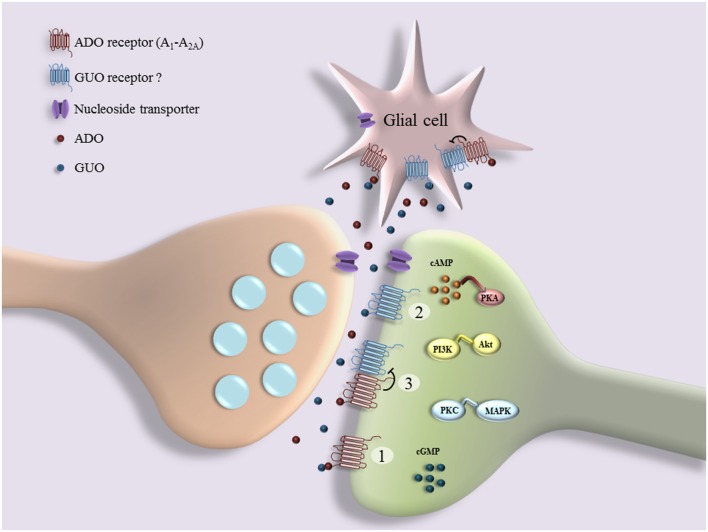
**Schematic representation of interplay between guanosine (GUO) and adenosine (ADO) binding to receptors: GUO binds to ADO receptor (A_1_R and A_2A_R) in competitive manner with ADO (1); GUO binds to putative unknown GUO receptor (2), which may form heterocomplexes with ADO receptors (3)**. Several effects of GUO are dependent on cell signaling pathways downstream of receptors (e.g., cAMP/Protein Kinase A (PKA), PI3K/Akt, PKC/MAPK, and cGMP), culminating in functional cell responses such as cell proliferation, survival, neuritogenesis, neuroprotection, and cell migration.

In addition to the above-mentioned data showing the existence of a putative unknown GPCR for GBPs, other findings also indicate that GUO may signal through A_1_R and/or A_2A_R (**Figure 1**). For example, it has been observed that GUO protects hippocampal slices against oxidative and inflammatory processes in an A_1_R dependent manner, while GUO-induced neuroprotection and stimulation of glutamate uptake involves A_2A_R activation (Dal-Cim et al., 2013). A_1_R blockade or A_2A_R activation can reverse GUO-evoked neuroprotective effect, suggesting that GUO effect may involve an interaction with A_1_R-A_2A_R oligomers (Lanznaster et al., 2016). Furthermore, GUA inhibition of renal Na^+^-ATPase activity, which is dependent on the activation of a Gi-coupled receptor, is blocked by antagonists of A_1_R (Wengert et al., 2011). In addition, while P2 receptor antagonists significantly reduce the stimulation of cell proliferation induced by GTP, a preferential A_2B_R antagonist only partially decreases the mitogenic activity of GUO in astrocytes (Ciccarelli et al., 2000). Overall, these data could suggest that GUO, probably with low affinity, may also bind to ADO receptors and act as an agonist, triggering alternative pathways to those promoted by ADO. However, it is needed to say that GUO binding to ADO receptors cannot account for all GUO-mediated effects in brain, since many of them still persist in the presence of both P1 and/or P2 receptor antagonists (Gysbers and Rathbone, 1992; Tasca and Souza, 2000; Frizzo et al., 2001). Indeed, the neuritogenic activity of GTP is not reproduced by ADA nucleotides, whereas the GUO effect is synergistic with that produced by A_1_R/A_2_R agonists and it is P1 receptors independent, since it is not inhibited by A_1_R/A_2_R antagonists (Gysbers and Rathbone, 1996a). Overall, these results support either the existence of specific GPCRs for GUO and/or of receptor heterocomplexes between GUO and ADO receptors (**Figure 1**). This functional interplay between GUO and ADO suggests that putative GUO receptors might share some particular features, yet elusive to current experimental approaches, with ARs, or alternatively that they might form heterocomplexes with ARs modulating the reciprocal activity (**Figure 1**). Finally, an additional theory, according to the hypothesis previously reported (Ciruela, 2013), consists of the no existence of putative GUO receptors, thus this molecule would signal through existing receptor complexes containing A_1_R and/or A_2_R (**Figure 1**). The elucidation of the mechanism of GUO and ADO receptors interaction and the characterization of membrane-binding sites are under current investigation in our laboratory. Overall, these possibilities may partially explain as to why GUO receptors have not still been identified, but they may also spur research to explore among both cloned known (i.e., ARs) and orphan GPCRs to find a potential specific GUO receptor. To this aim, good GPCR candidates to explore as potential GUO receptors could be GPR174/LPS3 (Civelli et al., 2013), showing high homology with P2Y10, and GPR23/LPA4, which shares high homology with human P2Y5 receptor and seems to be a potential putative GUO receptor, as suggested by preliminary data. (Di Liberto et al., 2012; Lanznaster et al., 2016). In addition, based on the evidence that ADO receptors, mainly A_1_R and A_2_R, form heteroreceptor complexes with several members P2Y receptor family (Borroto-Escuela et al., 2014) and on the high-sequence homology between GPR23 and P2Y5 receptors, it is reasonable to hypothesize an interaction between GPR23 and ADO receptors.

## GBPs and Cell Signaling

As stated above, several effects of GUO are mediated through the binding to unknown GPCRs, and therefore dependent on cell signaling pathways (Gysbers and Rathbone, 1996a,b; Traversa et al., 2003). Indeed, extracellular GUO enhances the neuritogenic effects of nerve growth factor (NGF) on PC12 cells through both cAMP-dependent (Gysbers and Rathbone, 1992; Gysbers and Rathbone, 1996a,b; Bau et al., 2005), and cAMP-independent mechanisms, such as the stimulation of soluble guanylate cyclase and the increase of intracellular cyclic GMP (cGMP; Bau et al., 2005). GUO is also able to stimulate, *in vitro*, neural stem cell proliferation by activating the cAMP response element binding (CREB) pathway (Su et al., 2013), whereas it protects hippocampal neurons against glutamate-induced cell death by a mechanism that involves the Phosphoinositide 3-kinase (PI3K)/Protein kinase B (PKB/Akt)/Glycogen Synthase Kinase3β (GSK3β) pathway (Molz et al., 2011). Similarly, in C6 glioma cells, GUO protects against 6-hydroxydopamine (6-OHDA)-induced neurotoxicity through the activation of the survival pathways regulated by extracellular signal-regulated kinases (ERK) and PI3K/Akt (Giuliani et al., 2015). As well, GUO is protective in different *in vitro* models against hypoglycemia by modulating oxidative and nitrosative stress and astroglial responses, such as glutamate uptake through different signaling involving protein kinase C (PKC), PI3K, p38, and ERK pathways (Quincozes-Santos et al., 2013). Additionally, GUO prevents in hippocampal slices mitochondrial membrane depolarization, inhibits (NF-kB) activation and reduces inducible nitric oxide synthase (iNOS) and ROS production via PI3K and mitogen-activated protein kinases (MAPK)/ERK (Dal-Cim et al., 2013). Also, it is neuroprotective against mitochondrial oxidative stress in neuroblastoma SH-SY5Y cells via PI3K/Akt/GSK-3β pathway (Dal-Cim et al., 2012). Again, anti-inflammatory effects of GUO on microglia are mediated by activation of the PI3K/Akt/MAPK (ERK1/2 and p38) pathways (D’Alimonte et al., 2007). Finally, GUO, even administered *in vivo* for a systemic treatment, is able to produce antidepressant-like effects, dependent on the modulation of NMDA receptors and mediated by cGMP and PI3K/mammalian target of rapamycin (mTOR) pathways (Bettio et al., 2012, 2014). Taken together, these data generated in different experimental models, mainly in *in vitro* studies, point out intracellular signaling events promoted by GUO and involving cAMP pathway as well as those linked to the PI3K and MAPK system. All these evidences may indirectly support the possibility that GBPs, particularly GUO, may activate cell surface receptors.

## GBPs and Neuroprotection

Many studies have demonstrated that GBPs are able to exert neuroprotective effects on a wide variety of cell cultures and *in vivo* models (Schmidt et al., 2007; Ribeiro et al., 2015). These effects would be elicited through three main actions: by counteracting glutamate excitotoxicity; by preventing or attenuating neuroinflammation; by counteracting oxidative stress. In this part of the review we summarize recent research data investigating the neuroprotective effects of GBPs. Of note, we are not dealing here with the scarce clinical therapeutic available data.

### Neuroprotection against Glutamate Toxicity

Different *in vivo* ischemic models have been used to show the neuroprotective role of GBPs, including oxygen and glucose deprivation, hypoxia models and *in vivo* transient and permanent ischemic stroke (Ribeiro et al., 2015). Generally, in ischemia conditions, glutamate released from neurons and astrocytes in the extracellular space, together with the ischemia-induced neuronal depolarization, leads to an abnormal neuronal firing and increased intracellular Ca^2+^ concentration with a subsequent loss of synaptic function and cell death (Brassai et al., 2015). In these models, the neuroprotective action of GBPs against glutamate toxicity and related intracellular signaling has been extensively characterized. In such way, it has been reported that GUO nucleotides, such as GTP, GDP and GMP, are able to inhibit NMDA-induced neurotoxicity in cultured hippocampal and neocortical neurons (Morciano et al., 2004), and that GMP is neuroprotective against glutamate or oxygen/glucose deprivation-induced neurotoxicity and against NMDA-induced apoptosis in hippocampal slices (Molz et al., 2005, 2008; Chang et al., 2008). Furthermore, in hippocampal slices and SH-SY5Y cells exposed to oxygen/glucose deprivation, GUO is neuroprotective against glutamate induced excitotoxicity via PI3K/Akt/GSK3β pathway and attenuates glutamate uptake impairment by promoting the activation of K^+^ channels and activating *G*_i_/*G*_o_-proteins-coupled signaling (Dal-Cim et al., 2011, 2012, 2013). In addition, there is evidence that GUO is effective in counteracting the glutamate toxicity, thus preventing most of the effects promoted by hyperprolinemia, such as alteration of glutamatergic homeostasis due to a reduction of glutamate uptake and coupled to a decrease of membrane Na^+^, K^+^-ATPase activity and of intracellular ATP levels (Ferreira et al., 2012). As well, GUO increases glutamate uptake in hippocampal slices of neonatal rats exposed to a hypoxic-ischemic insult (Moretto et al., 2009). Similarly, it has been shown that GUO protects hippocampal neuron against ischemia induced by bilateral common carotid artery occlusion (Ganzella et al., 2012), decreases infarct volume and improves neurological function following ischemic stroke in rats (Rathbone et al., 2011). Also, GUO has been shown to protect neurons against cortical focal ischemia (Hansel et al., 2014, 2015) and prolong rat survival by decreasing neurological deficits following transient middle cerebral artery occlusion (Chang et al., 2008; Connell et al., 2013). Finally, GUO shows marked anticonvulsant/antiepileptic effects in several models of epilepsy (Kovacs et al., 2015a). For example, GUO and other GBPs attenuate the lipopolysaccharide-evoked increase in spike-wave discharges number in rats (Kovacs et al., 2015a,b). Also, GBPs are able to prevent seizures (de Oliveira et al., 2004; Schmidt et al., 2005) or to counteract electrophysiological spectral changes, including glutamate store into synaptic vesicles, in a quinolinic acid-induced seizure model (Tavares et al., 2005, 2008; Torres et al., 2010). Taken together, the reported data indicate that GBPs exert a neuroprotective action against glutamate toxicity and that many of the related effects on the glutamatergic system are produced by increasing astrocytic glutamate uptake (de Oliveira et al., 2004; Frizzo et al., 2005; Moretto et al., 2005; Schmidt et al., 2007), thus avoiding excitotoxic glutamate accumulation (Deutsch et al., 2008). Since this neuroprotective mechanism is largely accepted, it has been suggested the development of GBPs for the treatment of disorders associated with glutamate excitotoxicity (Deutsch et al., 2005).

### Neuroprotection against Neuroinflammation-Induced Neuronal Damage

Neuroinflammation is known to contribute to neuronal damage in different noxious brain conditions (Allan and Rothwell, 2003). Thus, the GBPs role in preventing or attenuating neuroinflammation has been extensively studied. In this context, GUO is able to prevent lipopolysaccharide-induced pro-inflammatory response and oxidative stress in hippocampal primary astrocytes through activation of the Heme Oxygenase-1 (HO-1) pathway and independently of ADO system (Bellaver et al., 2015). Also, in focal cerebral ischemia model, GUO administration reduces pro-inflammatory and increases anti-inflammatory cytokine levels with reduction of microglial/macrophage cell number, leading to a decrease in neuronal damage and infarct volume with a positive effect in functional recovery (Hansel et al., 2015). Additionally, GUO has been shown to be neuroprotective in an ischemic stroke model, by inhibiting proinflammatory interleukin-8 (Connell et al., 2013), and to be able to protect hippocampal slices against inflammatory processes induced by combined glucose and oxygen deprivation, via a mechanism that involves MAPK/ERK and A_1_R activation (Dal-Cim et al., 2013). Finally, systemic administration of GUO following spinal cord injury in rats significantly improves motor and sensory functions, in association with attenuation of the inflammatory response and apoptotic cell death, and with an increase in sparing of axons and myelin preservation (Jiang et al., 2008a). Taken together, the neuroprotective effects exerted by GBPs against neuroinflammation support the existence of a combined activity of these compounds on both neurons and glial cells, particularly microglia cells, as already well shown for ABPs.

### Neuroprotection against Reactive Oxygen Species

Oxidative stress can be defined as an increase of pro-oxidative mechanisms due to a disturbance of the equilibrium between pro-oxidant and anti-oxidant systems, which leads to the production of peroxides and free radicals that damage all the cell components, including proteins, lipids, and DNA (Kohen and Nyska, 2002). A large number of experimental data support a sustained role of GBPs in neuroprotection against oxidative stress in different *in vivo* and *in vitro* models. For instance, in cortical brain slices, GUO is neuroprotective against methylmercury-induced oxidative stress (Roos et al., 2009), and it is also able to prevent the increase in oxidative damage as well as in the levels of glutamate in a rat model of chronic hepatic encephalopathy (Paniz et al., 2014). In C6 glioma cells, GUO is neuroprotective against hypoglycemia by modulating oxidative and nitrosative stress and astroglial responses, such as glutamate uptake, glutamine synthetase activity and glutathione levels (Quincozes-Santos et al., 2013). Similarly, GUO protects SH-SY5Y neuroblastoma cells against β-amyloid toxicity, by decreasing the formation of early ROS (Pettifer et al., 2004; Tarozzi et al., 2010) and against the mitochondrial oxidative stress, induced by the blockage of mitochondrial complexes, by inducing HO-1 via PI3K/Akt/GSK-3β (Dal-Cim et al., 2012). Also, in hippocampal slices GUO reduces ROS production, prevents mitochondrial membrane depolarization, inhibits NF-kB activation and reduces iNOS following oxygen/glucose deprivation (Dal-Cim et al., 2013). GUO neuroprotection against glutamate-induced cell death mediated by the inhibition of iNOS has been further documented in hippocampal neurons (Molz et al., 2011). GUO may also be effective in attenuating the MPP^+^-induced collapse of mitochondrial transmembrane potential and in preventing the subsequent activation of caspase-3, thus protecting dopaminergic neurons against mitochondrial stress-induced damage (Li et al., 2014). In addition, in *in vivo* studies, GUO is able to reduce hippocampal oxidative damage in rats submitted to sepsis by cecal ligation and perforation (Petronilho et al., 2012), in which it also causes a recovery of memory impairment, and in mice submitted to acute restraint stress, in which GUO produces antidepressant-like effects (Bettio et al., 2012). Furthermore, in focal cerebral ischemia model, GUO administration reduces ROS levels and increases superoxide dismutase levels in the brain, leading to a decrease in neuronal damage and infarct volume with a positive effect in functional recovery (Hansel et al., 2015).

Taken together all the described findings strongly suggest a relevant neuroprotective role of GBPs in the mammalian brain, providing new targets and strategies for the treatment of brain diseases.

## Behavioral Response to GBPs

Guanine-based purine effects on behavioral response have been investigated with the limit imposed by the lack of specific receptors and thereby available antagonist compounds. Noteworthy, systemic treatments show that GUO, even at high doses, does not induce mortality and any obvious behavioral disturbances, such as alterations of coordination, locomotion, body weight, and core temperature. Additionally, GUO does not produce depressant activity but rather excitant effects that resembles those caused by some ARs antagonists (El Yacoubi et al., 2003; Vinade et al., 2003; Schmidt et al., 2010). On the other hand, in memory tests in rats, GUO pre-training administration is amnesic on inhibitory avoidance task (Roesler et al., 2000; Vinade et al., 2003, 2004, 2005; Saute et al., 2006). This amnesic effect is compatible with inhibition of glutamatergic system and seems to be independent from A_1_R and A_2_R activation, since it is not inhibited by the ADO receptor antagonist caffeine (Vinade et al., 2004, 2005). This GUO effect on memory is also present after chronic administration with anticonvulsant doses of this nucleoside and, again, it is not blocked by ADO receptor antagonists (Vinade et al., 2003, 2004). Additionally, GMP is able to counteract the facilitatory effect of post-training intra-hippocampal glutamate administration on inhibitory avoidance task (Rubin et al., 1996). In contrast, in a different memory model, GUA seems to prevent the amnesic effect caused by L-NG-nitro-L-arginine methyl ester (L-NAME), an inhibitor of NOS (Giuliani et al., 2012b).

Regarding studies concerning motor behavior, it has been shown that GUO does not modulate spontaneous locomotion (Tort et al., 2004), but decreases locomotor activity in the open field test (Vinade et al., 2005). Also, it has been described that GUO improves motor behavior both in rats with parkinsonism, by decreasing bradykinesia (Su et al., 2009), and in rats with spinal cord injury (Jiang et al., 2007). Interestingly, GUO is able to produce antidepressant-like effects evaluated by means of the forced swimming test and the tail suspension test in mice. This behavioral effect would be dependent on the modulation of NMDA receptors, nitric oxide-cGMP and PI3K/mTOR pathways (Bettio et al., 2012, 2014). Additionally, systemic administration of GMP induces anxiolytic-like behavior in rats (Almeida et al., 2010). This anxiolytic-like effect of GBPs is further supported by data showing that chronic administration of GUO in mice exhibits an anxiolytic effect in the hole board task (Vinade et al., 2003), in the tail suspension test and in the open field test (Bettio et al., 2016). Similarly, acute GUO administration has also been shown to induce robust anxiolytic-like effects. Of note, pretreatment with caffeine completely abolishes GUO anxiolytic effects (Almeida et al., 2016). Noteworthy, preliminary experiments in our laboratory confirm the anxiolytic effect of GUO in three different behavioral tests in rats: light/dark, elevated plus-maze and open field (personal observations). All these behavioral data support an anxiolytic effect of GBPs, in particular of GUO, which is opposite to the depressant action of AR activation (Krugel, 2015). However, in several of the reported investigations, it is not clear the A_1_R and A_2A_R involvement in the behavioral outcomes following GUO treatment (Roesler et al., 2000; Vinade et al., 2004). Therefore, this issue needs further investigations, which should take into account other possibilities (*see* the GBPs receptor section, e.g., GUO may bind to heteroreceptor complexes).

## Neuronal Plasticity Mediated by GBPs

To deal with the effects of GBPs on neural plasticity we have grouped the existing experimental data in the following four subjects: cell proliferation; neurite growth; synaptogenesis and synaptic activity; and synaptic plasticity.

### Cell Proliferation

Cell proliferation mediated by GBPs has been largely investigated in glial cells and in a lesser extent in neuronal cells. Concerning this latter aspect, it has been shown that GBPs exert antiproliferative effects in human SH-SY5Y neuroblastoma cells by inducing a S-phase cell-cycle arrest, with up-regulation of genes for S-phase entry (Cyclin E2) and down-regulation of genes promoting cell-cycle progression, such as the cyclin B1/B2 that prevents S-phase exit (Guarnieri et al., 2009). By contrast, GUO is able to stimulate *in vitro* neural stem cell proliferation by activating the cAMP-CREB pathway (Su et al., 2013) and to potentiate neurogenesis in the subventricular zone in a rat model of parkinsonism (Su et al., 2009). Chronic treatment of mice for 21 days with GUO results in a significant increase in the number of immature neurons in the ventral hippocampal, which is known to regulate emotional and motivational behaviors (Bettio et al., 2016). Finally, GMP or GUO treatment of co-culture cerebellar neurons/astrocytes would not induce proliferation of neurons or astrocytes although it promotes MAPK activation (Decker et al., 2007).

Differently from data available on neurons, evidences pointing to mitogenic effects of GBPs on astrocytes are broad. The first result, already published in 1991, showed that GUO, GMP, GDP and GTP are able to increase cAMP and to stimulate astroblast proliferation through, at least in part, the enhancement of ADO extracellular levels (Kim et al., 1991; Rathbone et al., 1991; Ciccarelli et al., 2000). In this context, GUO has also been shown to stimulate astrocytes proliferation by producing large amounts of neuroprotective factors (Rathbone et al., 1999; Ciccarelli et al., 2001). Also, it has been observed that the mitogenic effects of GUO on astrocytes is significantly enhanced by the co-presence in the culture of microglial cells releasing soluble factors (Ciccarelli et al., 2000). Additionally, after spinal cord injury, chronic treatment with GUO increases remyelination by increasing proliferation and differentiation of endogenous adult oligodendroglial progenitor cells (Jiang et al., 2008a). In contrast with data suggesting a positive role of GBPs in the regulation of glial cell proliferation, Garozzo et al. (Garozzo et al., 2010) have demonstrated that GBPs (GUO, GMP, and especially GUA) exert a marked growth inhibition in standard cell culture conditions, on the U87 and U373 glioma cell lines and on other cancer cell lines, by decreasing the rate of progression through the S-phase. Interestingly, this antiproliferative effect is antagonized by ABPs and it is in part dependent on the intracellular hypoxanthine-guanine phosphoribosyltransferase, an enzyme responsible for the conversion of GUA into GMP, suggesting the central role of the intracellular metabolism of GUA for these inhibitory effects on cell proliferation (Garozzo et al., 2010). A similar antiproliferative effect has been observed in human SH-SY5Y neuroblastoma cells following GTP or GUO treatment (Guarnieri et al., 2009). Taken together, the opposite effects of GUO on cell proliferation may be dependent on the experimental model and cell lines used, which subsequently can lead to the activation of different GBPs binding sites or receptor–receptor interactions, and consequently to different functional outcomes. Nevertheless, taking into consideration that GBPs exert antiproliferative effects in brain cancer cell lines, it would be relevant to underline the possible anti-tumoral effect exerted by GBPs. In our opinion, this aspect deserves further investigation.

### Neurite Outgrowth

GBPs are able to play significant neurotrophic roles by stimulating synthesis and secretion of trophic factors, in both neuronal and glial cells, and also by inducing cell differentiation, including neuritogenesis (Schmidt et al., 2007; Rathbone et al., 2008). Extracellular GUO not only stimulates neurite outgrowth in primary cultures of rat hippocampal neurons and in pheochromocytoma PC12 cells (Gysbers and Rathbone, 1996b; Rathbone et al., 1999), but also it enhances the neuritogenic effects of NGF on PC12 cells through both cAMP-dependent and -independent mechanisms (Gysbers and Rathbone, 1996a,b; Bau et al., 2005). In the same cell culture model, GTP may also enhance, by increasing intracellular Ca^2+^, NGF-dependent neurite outgrowth (Gysbers and Rathbone, 1996a; Gysbers et al., 2000). In human SH-SY5Y neuroblastoma cells, both GUO and GTP induce an increase in the number of neurites as well as in neurite length, and they also promote cell differentiation (Guarnieri et al., 2009). Finally GUO, although in a less extent than ADO, counteracts axonal degeneration following axotomy in cultured dorsal root ganglia neurons (Press and Milbrandt, 2009). All these effects on neurites growth are probably linked to a GBPs direct role in the regulation of neurotrophic factors expression and release (Schmidt et al., 2007; Rathbone et al., 2008). Indeed, GUO and GTP enhance in astrocytes cultures the synthesis and release of NGF, fibroblast growth factor-2 and transforming growth factor β (Middlemiss et al., 1995; Rathbone et al., 1999; Ciccarelli et al., 2001; Schmidt et al., 2007).

### Synaptogenesis and Synaptic Activity

In addition to neuritogenesis, GUO may play a role in synaptogenesis. In fact, *in vivo* administration of GUO to the rat visual cortex promotes an increase in the number and size of synaptic buttons along the axonal branches projecting from the rat visual cortex to other cortical areas and to subcortical structures (Gerrikagoitia and Martinez-Millan, 2009). Interestingly, GUO may also increase cholesterol effiux and apolipoprotein E expression in astrocytes, through the PI3K/ERK1/2 pathways (Ballerini et al., 2006a), which have been demonstrated to promote the development of new synapses in retinal ganglion cell cultures (Goritz et al., 2005). We think that this interesting GBPs effect on synaptogenesis deserves more attention and more molecular approaches.

Concerning GBPs role in neuronal synaptic activity, it is known that GTP is stored in synaptic vesicles and co-released with ATP by exocytosis (Wagner et al., 1978; Santos et al., 2006). Also, indirect evidences indicate that GUO can be released from neurons following depolarization (Fredholm and Vernet, 1979). However, as already stated above, glial cells are the principal source of extracellular GUO, in both physiological and pathological conditions (Ciccarelli et al., 1999). GUO may also accumulate extracellularly as a result of both metabolism of extracellular nucleotides, by ectonucleotidases, and transport from the cytoplasm through the bi-directional nucleoside transporters (Schmidt et al., 2007). Once in the extracellular space, GBPs may interact with neurons at the synaptic level. However, the role of GBPs, both as neurotransmitters or neuromodulators, is poorly understood due to the above-mentioned fact that their putative own receptors are still unknown. Nevertheless, GBPs appear to interfere with prejunctional acethylcoline release in the circular muscle layer of mouse colon, and their effects are dependent on their cellular uptake and independent from ABP receptors (Zizzo et al., 2011). Furthermore, GUO is able to induce muscular relaxation; this effect is dependent on GUO cellular uptake, adenylyl cyclase activation and increase in cAMP intracellular levels, while it is independent of neural action potentials, ARs and K^+^ channel activation (Zizzo et al., 2013). In several experimental conditions GBPs seem to modulate glutamatergic neurotransmission. Indeed, GBPs prevent cell responses to excitatory amino-acids by blocking glutamate and AMPA binding in membrane preparations without interacting with G-proteins (Souza and Ramirez, 1991; Paz et al., 1994; Tasca et al., 1995, 1998; Dev et al., 1996; Migani et al., 1997; Porciuncula et al., 2002). Also, GBPs may inhibit membrane ion currents induced by NMDA (Paas et al., 1996) or kainate (Rubin et al., 1996; Poulopoulou and Nowak, 1998). These effects suggest that GBPs may exert a competitive inhibitory mechanism, acting as antagonists of ionotropic glutamate receptors, thus antagonizing glutamatergic neurotoxicicity, as already discussed in a previous section. Furthermore, GBPs show anti-epileptic activity not only by inhibiting lipopolysaccharide-evoked increase in spike-wave discharges (Kovacs et al., 2015a,b) but also by counteracting electrophysiological spectral changes, including glutamate storage into synaptic vesicles (Tavares et al., 2005, 2008; Torres et al., 2010), and by preventing seizures (de Oliveira et al., 2004; Schmidt et al., 2005).

On the other hand, there are evidences supporting that, at high doses, GMP may induce activation of ionotropic glutamate receptors and inhibition of glutamate transporters activity (Molz et al., 2009). Furthermore, antinociceptive effects have been observed after systemic administration of GUO, which would be linked to the modulation of non-NMDA glutamate receptors, either by means of a direct interaction with receptors or with their signal transduction mechanisms (Schmidt et al., 2008, 2010). Finally, GUO has also been shown to modulate glutamate transporters activity by decreasing glutamate uptake into synaptic vesicles. Therefore, it would modulate the amount of transmitter inside the vesicles, which might influence synaptic strength as well as vulnerability to neural damage processes, by decreasing the amount of glutamate released to the synaptic cleft (Tasca et al., 2004). Overall, the above-mentioned works largely support the modulatory role of GBPs at the glutamatergic synaptic level. However, further investigations are needed, in particular regarding the modulation of other neurotransmitters by GBPs.

### Synaptic Plasticity

In contrast to the large body of data existent supporting the regulation exerted by ABPs on synaptic plasticity through their broad family of receptors, very little is known, at present, about the GBPs role in synaptic plasticity. The principal reason of this limitation is consists, as broadly repeated, of the lack of known receptor(s). Consequently, it has not been possible the discovery and use of selective agonists/antagonists. Importantly, these compounds would be crucial for determining the exact role of GBPs in various body compartments, including the CNS. Nevertheless, despite this limitation, it has been possible to assess, as described above, the modulation of glutamatergic neurotransmission by GBPs in several experimental conditions, which is known to be relevant in synaptic plasticity. Noteworthy, the effects of GBPs on glutamatergic system are mainly attributed to the increase in astrocytic glutamate uptake, or to interference with the function of ionotropic glutamate receptors. However, no data is available supporting direct neuronal presynaptic modulation of glutamate release. Indeed, GBPs block glutamate and AMPA binding in membrane preparations (Souza and Ramirez, 1991; Paz et al., 1994; Tasca et al., 1995, 1998; Dev et al., 1996; Migani et al., 1997; Porciuncula et al., 2002). Also, GBPs inhibit membrane currents induced by NMDA (Paas et al., 1996) or kainate (Rubin et al., 1996; Poulopoulou and Nowak, 1998), as well as prevent seizures (de Oliveira et al., 2004; Schmidt et al., 2005). Finally, it has also been shown that GMP, at high doses, induce activation of ionotropic glutamate receptors (Molz et al., 2009). Taken together, the actual experimental data suggest that GBPs may play a role on synaptic plasticity by modulating, in different ways, the glutamatergic system.

## Conclusion and Outlook

In the present article, we have extensively summarized the present data concerning the role of the GBPs system in regulating neuronal function and plasticity. On the other hand, we have also briefly discussed the scarce knowledge regarding the mechanisms of action of these orphan neuromodulators. However, we would not like to simply conclude with the habitual sentence, classically reported in previous reviews on this issue, encouraging the scientific community to advance in GBPs research. Thus, it is clear that further work is still necessary to elucidate the mechanisms of action of GBPs, this is, the cloning of specific receptors and characterization of second messengers related to their extracellular effects. But this review has been conceived and carried out with the purpose of acquiring a new consciousness. We honestly think that research aiming the identification of GBPs receptors may be based on a recent suggestion (Ciruela, 2013) and our preliminary data, evidencing that GUO is somehow linked to ARs. Thus, cooperative efforts should be directed to explore both new GBPs putative receptors and their possible interplay, especially in terms of oligomerization and/or allosteric modulation, with ARs, and also the possible direct binding of GBPs to ARs. Overall, a very exciting work is expected to be completed, in a cooperative and collaborative way, in the next years in order to fill this “big gap” in the purinergic transmission field.

## Author Contributions

BN, CF, CAF, CDF: contributed to plan the review and to revision of manuscript DLV, CR, MG: contributed to search for specific references and prepare the figure BN, CR, MG, DLV, CF: contributed to write the review BN, CDF, MG, CR, DLV, GR, FM, FDV, DIP: contributed to experimental data reported in the review.

## Conflict of Interest Statement

The authors declare that the research was conducted in the absence of any commercial or financial relationships that could be construed as a potential conflict of interest.
